# Cranberry proanthocyanidins inhibit the adherence properties of *Candida albicans *and cytokine secretion by oral epithelial cells

**DOI:** 10.1186/1472-6882-12-6

**Published:** 2012-01-16

**Authors:** Mark Feldman, Shinichi Tanabe, Amy Howell, Daniel Grenier

**Affiliations:** 1 Groupe de Recherche en Écologie Buccale, Faculté de Médecine Dentaire, Université Laval, Quebec City, Quebec, Canada; 2 Marucci Center for Blueberry and Cranberry Research, Rutgers, The State University of New Jersey, Chatsworth, New Jersey, USA

## Abstract

**Background:**

Oral candidiasis is a common fungal disease mainly caused by *Candida albicans*. The aim of this study was to investigate the effects of A-type cranberry proanthocyanidins (AC-PACs) on pathogenic properties of *C. albicans *as well as on the inflammatory response of oral epithelial cells induced by this oral pathogen.

**Methods:**

Microplate dilution assays were performed to determine the effect of AC-PACs on *C. albicans *growth as well as biofilm formation stained with crystal violet. Adhesion of FITC-labeled *C. albicans *to oral epithelial cells and to acrylic resin disks was monitored by fluorometry. The effects of AC-PACs on *C. albicans*-induced cytokine secretion, nuclear factor-kappa B (NF-κB) p65 activation and kinase phosphorylation in oral epithelial cells were determined by immunological assays.

**Results:**

Although AC-PACs did not affect growth of *C. albicans*, it prevented biofilm formation and reduced adherence of *C. albicans *to oral epithelial cells and saliva-coated acrylic resin discs. In addition, AC-PACs significantly decreased the secretion of IL-8 and IL-6 by oral epithelial cells stimulated with *C. albicans*. This anti-inflammatory effect was associated with reduced activation of NF-κB p65 and phosphorylation of specific signal intracellular kinases.

**Conclusion:**

AC-PACs by affecting the adherence properties of *C. albicans *and attenuating the inflammatory response induced by this pathogen represent potential novel therapeutic agents for the prevention/treatment of oral candidiasis.

## Background

*Candida albicans *is a commensal microorganism that colonizes the oral cavity of a large proportion of humans. Although in most cases this yeast does not cause any harmful effects, an overgrowth of *C. albicans *may result in candidiasis. Several factors that induce changes in the oral environment can predispose individuals to oral candidiasis and include: antibiotics and corticosteroid use, xerostomia, diabetes mellitus, nutritional deficiencies, and immunosuppressive diseases and therapy [1]. More specifically, denture stomatitis is a common form of candidiasis affecting denture wearers and characterized by an inflammation of the oral mucosal areas induced by *C. albicans *[2]. Several virulence properties of *C. albicans*, which contribute to the development of oral candidiasis have been identified. They include i) adhesins that allow these organisms to adhere to oral epithelial cells with subsequent invasion [3], ii) the capacity to form biofilm on both oral mucosa and denture devices [4,5], and iii) the ability to switch from yeast form to mycelium form [6].

In mucosal infections such as oral candidiasis, the innate immunity is very important and involves neutrophils, macrophages, natural killer cells, dendritic cells, and non-hematopoietic cells, such as mucosal epithelial cells. There are two main outcomes from the interaction of innate immune cells with *C. albicans*: i) a direct anti-fungal activity, and ii) a regulatory activity that promotes the chemotaxis, proliferation, and terminal differentiation of cells from both innate and adaptive immune systems, through the synthesis of cytokines. Although pro-inflammatory cytokines might serve to limit the progression of infection, they may also be involved in immunopathology and tissue destruction by inducing the secretion of host matrix metalloproteinases (MMPs) [7-10] or by provoking an uncontrollable leukocyte mobilization [11].

Despite the availability of a wide range of antifungal agents for the treatment of oral candidiasis, failure of therapy is observed frequently [12]. As a matter of fact, the ability of *C. albicans *to form biofilms on epithelial surfaces and prosthetic devices reduces its susceptibility to antifungal agents [13,14]. Cranberry extracts and purified compounds have been suggested as potential therapeutic agents in various areas of health, including cancer, cardiovascular diseases and infectious diseases [15-17]. More specifically, the proanthocyanidins isolated from cranberry fruits possessed unusual structures with A-type linkages containing a second ether linkage between an A-ring of the lower unit and the C-2 ring of the upper unit (O7 C2), a characteristic that has been associated with their anti-adherence property [18]. In this study we investigated the ability of A-type cranberry proanthocyanidins (AC-PACs) to inhibit growth, adherence properties and biofilm formation of *C. albicans*, as well as to reduce the inflammatory response of oral epithelial cells induced by *C. albicans*.

## Methods

### Isolation of A-type cranberry proanthocyanidins

Cranberry proanthocyanidins were isolated from cranberry fruit (*Vaccinium macrocarpon *Ait.) using solid-phase chromatography as described previously [19]. Briefly, cranberry fruit was homogenized with 70% aqueous acetone and filtered, and the pulp was discarded. The collected extract was concentrated under reduced pressure to remove acetone. The cranberry extract was suspended in water, applied to a preconditioned C_18 _solid-phase chromatography column, and washed with water to remove sugars, followed by acidified aqueous methanol to remove acids. The fats and waxes retained on the C_18 _sorbent were discarded. The polyphenolic fraction containing anthocyanins, flavonol glycosides, and proanthocyanidins (confirmed using reverse-phase high-pressure liquid chromatography [HPLC] with diode array detection) was eluted with 100% methanol and dried under reduced pressure. This fraction was suspended in 50% ethanol (EtOH) and applied to a preconditioned Sephadex LH-20 column which was washed with 50% EtOH to remove low-molecular-weight anthocyanins and flavonol glycosides. Proanthocyanidins adsorbed to the LH-20 were eluted from the column with 70% aqueous acetone and monitored using diode array detection at 280 nm. The absence of absorption at 360 nm and 450 nm confirmed that anthocyanins and flavonol glycosides were removed. Acetone was removed under reduced pressure and the resulting purified proanthocyanidin extract freeze-dried. The presence of A-type bonds and the concentration of proanthocyanidins in the preparation were evaluated using various analytical methods including ^13^C nuclear magnetic resonance (NMR), electrospray mass spectrometry, matrix-assisted laser desorption ionization-time-of-flight mass spectrometry, and acid-catalyzed degradation with phloroglucinol [18,20].

### *C. albicans *and culture conditions

*C. albicans *ATCC 28366 (oral origin) was cultivated in yeast nitrogen base (YNB) broth (BBL Microbiology Systems, Cockeysville, MD) + 0.5% glucose pH 7.0 under aerobic conditions at 37°C for 24 h. Cells were collected by centrifugation, washed twice with sterile physiologic saline (0.85% NaCl), concentrated ten times in saline and kept at 4°C until further use (for less than 6 days). Cells were diluted in YNB + 0.5% glucose pH 7.0 to the appropriate concentration just before to perform experiments.

### Effect on *C. albicans *growth

Serial 1:2 dilutions of AC-PACs in YNB broth + 0.5% glucose (from 100 to 6.25 μg/ml) were prepared in a flat-bottomed 96-well microplate (Sarstedt, Newton, NC). Control wells with no AC-PACs were also prepared. Then, an equal volume (100 μl) of the yeast suspension (5 × 10^4 ^cells /ml as determined with a Petroff-Hausser counting chamber) was added. After a 24-h incubation at 37°C under aerobic conditions, growth was monitored by recording the optical density at 660 nm. Assays were performed in triplicate and repeated three times.

### Effect on *C. albicans *biofilm formation

Two hundred and fifty μl of the yeast suspension (10^7 ^cells/ml) were added to wells of a 24-well tissue culture plate (Sarstedt) containing 250 μl of 1:2 serial dilutions (from 100 to 6.25 μg/ml) of AC-PACs in YNB broth + 0.5% glucose pH 7.0. Control wells with no AC-PACs were also inoculated. After incubation for 48 h at 37°C under aerobic conditions, spent media and free-floating microorganisms were removed by aspiration and the wells were washed twice with 10 mM phosphate-buffered saline (PBS, pH 7.4), prior to quantify biofilm by crystal violet staining, as previously reported [21]. Briefly, 0.02% crystal violet was added into wells for 45 min, which were then washed twice with PBS to remove unbound dye. After adding 250 μl of 95% ethanol into each well, the plate was shaken for 10 min to release the dye and the biofilm was quantified by measuring the absorbance at 550 nm (A_550_). Biofilm images of unstained preparations were acquired in phase-contrast mode using an Olympus FSX100 fluorescence microscope (Olympus, Tokyo, Japan). Assays were done in triplicate and three independent experiments were performed.

### Effect on *C. albicans *biofilm detachment

*C. albicans *biofilms were formed during 48 h as described above. After removing spent media and free-floating microorganisms and washing wells with PBS, biofilms were incubated with AC-PACs at concentrations ranging from 100 to 6.25 μg/ml (in YNB broth + 0.5% glucose) for 30 min and 120 min at 37°C. Control biofilms were incubated with YNB broth + 0.5% glucose alone. After incubation, biofilms were washed with PBS and quantified by crystal violet staining as described above. Assays were done in triplicate and three independent experiments were performed.

### Effect on adherence of *C. albicans *to oral epithelial cells

Human oral epithelial cells GMSM-K [22], kindly provided by Dr. Valerie Murrah (University of North Carolina, Chapel Hill, NC, USA), were cultured in Dulbecco's modified Eagle's medium (DMEM) supplemented with 10% heat-inactivated fetal bovine serum (FBS) and 100 μg/ml of penicillin G-streptomycin, and incubated at 37°C in an atmosphere of 5% CO_2_. Epithelial cells were seeded at a concentration of 4 × 10^5 ^cells/ml in above conditions in sterile 96-well clear bottom black microplates (Greiner Bio One, Frickenhausen, Germany) and incubated until confluence. Then, the wells were washed with DMEM-1% heat-inactivated FBS, blocked with 1% bovine serum albumin (BSA) to prevent non specific fungal attachment, and treated with AC-PACs diluted in DMEM-1% heat-inactivated FBS medium at concentrations ranging from 100 to 6.25 μg/ml for 1 h in a 5% CO_2 _atmosphere at 37°C. Control wells not treated with AC-PACs were also prepared. In parallel, cells from an overnight culture of *C. albicans *were suspended at 10^9 ^cells/ml in carbonate buffer (0.15 M NaCl/0.1 M Na_2_CO_3_, pH 9.0), and incubated for 1 h with continuous shaking with 0.1 mg/ml fluorescein isothiocyanate isomer I (FITC; Sigma-Aldrich Canada, Oakville, Ontario, Canada). *C. albicans *were then washed three times with PBS containing 0.05% Tween 20 and resuspended in PBS. FITC-labeled *C. albicans *were applied at a multiplicity of infection (MOI) of 15 (15 *C. albicans *per epithelial cell) to AC-PACs pre-treated or control epithelial cells and incubated for 30 min at 37°C. All incubation and washing steps were carried out in the dark. Following incubation, unbound *C. albicans *were aspirated and wells were washed three times with PBS. Adhered *C. albicans *were determined by monitoring the fluorescence using a Synergy 2 Multi-Mode Microplate Reader (BioTek Instruments, Winooski, VT, USA). The excitation and emission wavelengths were set at 488 and 522 nm, respectively. Image processing was performed using an Olympus FSX100 fluorescence microscope (Olympus). Images of adhered FITC-labeled *C. albicans *were observed at excitation and emission wavelengths of 488 and 522 nm, respectively, as well as in phase-contrast mode. The assays were performed in triplicate and repeated three times.

### Effect on adherence of *C. albicans *to acrylic resin disks

Acrylic resin disks (6 mm-diameter and 0.3 mm-thickness) were prepared as previously described [23], washed for two h in saline, and then autoclaved in saline. Non-stimulated saliva collected from five healthy volunteers was pooled, filtrated and inactivated at 60°C for 30 min. Acrylic resin disks were treated in the clarified heat-inactivated saliva for 1 h at 37°C with constant shaking and rinsed twice with PBS. Disks were incubated for 1 h at 37°C with intermittent shaking in the presence of equal volumes (100 μl) of FITC-labeled *C. albicans *(10^7 ^cells/ml) and AC-PACs at concentrations ranging from 100 to 6.25 μg/ml. Positive control consisted in disks incubated with FITC-labeled *C. albicans *in PBS but without AC-PACs. Unlabeled *C. albicans *incubated with discs served as negative control. Following incubation, unbound *C. albicans *were aspirated and disks were washed three times with PBS. Fluorescence was measured using a Synergy 2 Multi-Mode Microplate Reader. The excitation and emission wavelengths were set at 488 and 522 nm, respectively. Assays were performed in triplicate and repeated three times.

### Effect on cell surface hydrophobicity of *C. albicans*

This assay was performed according to the method described by Ishida et al. [24] and using xylene as organic solvent. Briefly, *C. albicans *at a concentration of 10^7 ^cells/ml was incubated for 30 min at 37°C with AC-PACs at 100 μg/ml. Yeast cells were then washed with PBS, suspended in the same buffer, and the optical density was determined spectrophotometrically at a wavelength of 660 nm. The cells were mixed with xylene (2.5:1, v/v), shaken for 2 min, and the tube was left for 20 min at room temperature in order to obtain separation of the phases. The turbidity of the aqueous phase was read at 660 nm. The hydrophobicity index (HI) was calculated as HI = (OD_control _- OD_test_) × 100/OD_control_, where OD_control _= optical density (660 nm) before xylene treatment and OD_test _= optical density (660 nm) after xylene treatment. Assays were performed in triplicate and repeated three times.

### Effect on the inflammatory response of oral epithelial cells stimulated with *C. albicans*

Human oral epithelial cells GMSM-K were seeded in a 12-well plate (4 × 10^5 ^cells/well in 1 ml) and cultured overnight in DMEM-10% heat-inactivated FBS medium containing antibiotics at 37°C in a 5% CO_2 _atmosphere to allow cell adhesion prior to the stimulation with *C. albicans*. The epithelial cells were pre-treated with increasing concentrations of AC-PACs (0, 25, 50, and 100 μg/ml) at 37°C in 5% CO_2 _for 1 h prior to stimulation with *C. albicans *at MOIs of 3 and 15. After a 6-h incubation with *C. albicans *at 37°C in 5% CO_2_, cell-free supernatants were collected and stored at -20°C until used. Commercial enzyme-linked immunosorbent assay (ELISA) kits (R & D Systems, Minneapolis, MN) were used to quantify interleukin-6 (IL-6) and interleukin-8 (IL-8) concentrations in the cell-free supernatants according to the manufacturer's protocols. The absorbance at 450 nm was read using a microplate reader with the wavelength correction set at 550 nm. The rated sensitivities of the commercial ELISA kits were 9.3 pg/ml for IL-6 and 31.2 pg/ml for IL-8. Assays were performed in triplicate and repeated three times.

### Analysis of NF-κB p65 activation and kinase expression

To identify the mechanism of action of AC-PACs on epithelial cells, their effect on different intracellular proteins associated with inflammation was investigated. Oral epithelial cells prepared as described above were incubated with 50 μg/ml of AC-PACs for 30 min and then stimulated with *C. albicans *at MOI of 15 for an additional 15 min at 37°C in a 5% CO_2 _atmosphere. Epithelial cells stimulated or not with *C. albicans *in the absence of AC-PACs served as controls. Whole-cell extracts were then prepared using nuclear extract kits (Active Motif, Carlsbad, CA) according to the manufacturer's protocol and adjusted to a protein concentration of 1 mg/ml. A sample of cell extracts were shipped frozen to SearchLight Protein Array Service (Pierce Biotechnology, Woburn, MA, USA) for the assay of four phosphorylated protein kinases [AKT (Ser473), AKT (Thr308), MEK1 (Ser217/Ser221), and ERK1/2 (Thr202/Tyr204)] that are involved in inflammatory signalling, by ELISA combined with piezoelectric printing technology. Nuclear factor-κB (NF-κB) p65, which is involved in secretion of proinflammatory mediators, was determined using a TransAm NF-κB p65 kit (Active Motif) according to the manufacturer's protocol. Assays were performed in triplicate and repeated three times.

### Effect of AC-PACs and *C. albicans *on viability of oral epithelial cells

Epithelial cells were grown until confluence in DMEM-10% heat-inactivated FBS medium supplemented with antibiotics at 37°C in a 5% CO_2 _atmosphere as described above. Epithelial cells were seeded at a concentration of 4 × 10^5 ^cells/ml in 96-well microplates and cultured until confluence. Then, cells were treated with increasing concentrations of AC-PACs (0, 6.25, 12.5, 25, 50 and 100 μg/ml), with *C. albicans *at MOIs of 3 and 15, or with both AC-PACs and *C. albicans*. After incubation for 24 h at 37°C in a 5% CO_2 _atmosphere, the cell viability was measured. Viability of epithelial cells was determined using a 3-[4, 5-dimethylthiazol-2-yl]-2, 5-diphenyltetrazolium (MTT) colorimetric assay (Roche Diagnostics, Mannheim, Germany) according to the manufacturer's protocol. This assay measures mitochondrial dehydrogenase activity. Assays were performed in quadruplicate and repeated three times.

### Statistical analysis

The means ± standard deviations were calculated. The statistical analysis was performed using Student t-test with a level of significance of *P *< 0.05.

## Results

**Figure 1:**
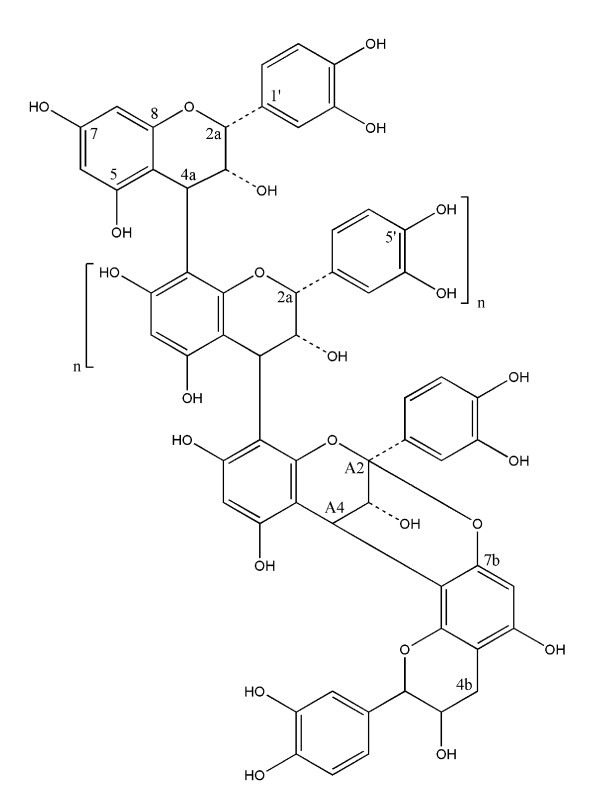
**Cranberry proanthocyanidins showing the presence of A-type linkages**.

**Figure 2:**
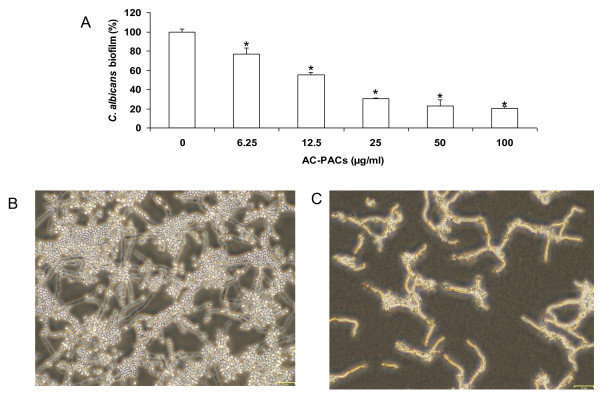
**Effect of AC-PACs on *C. albicans *biofilm formation**. Panel A: *C. albicans *biofilms were quantified by staining with crystal violet. Assays were done in triplicate and the means ± SD from three independent experiments were calculated. A value of 100% was assigned to the biofilm formed in the absence of AC-PACs. *, significantly lower than the value for the untreated control (*P *< 0.05). Panels B and C: Phase contrast microscopy of biofilms formed in the presence (B) or absence (C) of AC-PACs (25 μg/ml).

**Figure 3:**
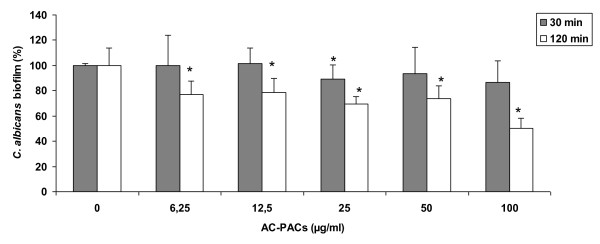
**Effect of AC-PACs on *C. albicans *biofilm desorption**. Newly formed biofilms (48 h) of *C. albicans *biofilms were treated (30 and 120 min) with AC-PACs prior to determine biofilm biomass by staining with crystal violet. Assays were done in triplicate and the means ± SD from three independent experiments were calculated. A value of 100% was assigned to the preformed biofilm unexposed to AC-PACs. *, significantly lower than the value for the unexposed control (*P *< 0.05).

The proanthocyanidin fraction purified from cranberry was characterized by ^13^C NMR. As shown in Figure 1, the proanthocyanidin molecules consist of epicatechin units with degrees of polymerization (DP) mainly of 4 and 5 containing at least one A-type linkage, as previously reported [18] (Figure 1). In order to investigate the capacity of AC-PACs to alter the virulence properties of *C. albicans*, we tested their effect on growth, biofilm formation and adhesion to oral epithelial cells and acrylic resin disks. At all tested concentrations, AC-PACs did not affect the growth of *C. albicans*. However, the biofilm of *C. albicans *formed after a 48 h-growth was significantly inhibited by AC-PACs in a dose-dependent manner (Figure 2A). At the lowest concentration tested (6.25 μg/ml), AC-PACs reduced biofilm formation by 23% ± 2.9%, while at 100 μg/ml the inhibition reached 80% ± 4.8% compared to untreated control (Figure 2A). The phase-contrast images clearly showed a marked reduction of biofilm as well as an alteration in its architecture when *C. albicans *was grown in the presence of 25 μg/ml of AC-PACs (Figure 2B) as compared to control (Figure 2C). Thereafter, the ability of AC-PACs to cause desorption of a preformed (48 h) biofilm of *C. albicans *was evaluated. A 30-min treatment of a newly formed biofilm with AC-PACs did not affect significantly their biomass. However, increasing the exposure time of *C. albicans *biofilms to AC-PACs at 120 min resulted in a significant detachment. AC-PACs at a concentration ranging from 6.25 to 50 μg/ml were able to reduce preformed biofilms by 25-30%, while the highest concentration (100 μg/ml) caused a 50% ± 8% desorption of *C. albicans *biofilm (Figure 3).

**Figure 4:**
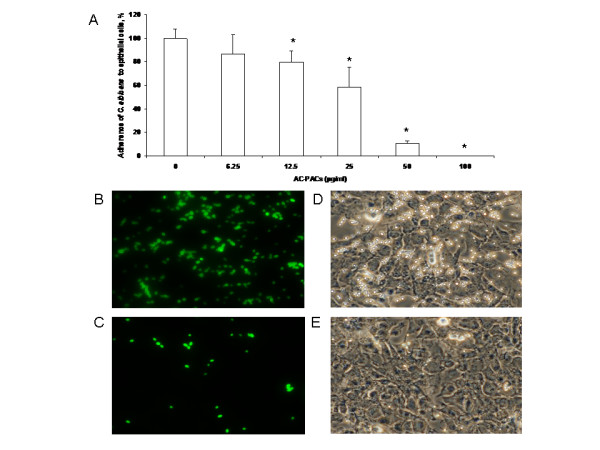
**Effect of AC-PACs on adherence of *C. albicans *to oral epithelial cells**. Panel A: FITC-labeled *C. albicans *adhered to epithelial cells were quantified by measuring fluorescence in a microplate reader. Assays were done in triplicate and the means ± SD from three independent experiments were calculated. A value of 100% was assigned to *C. albicans *adhered to epithelial cells not treated with AC-PACs. *, significantly lower than the value for the untreated control (*P *< 0.05). Panels B to E: Image processing was performed using fluorescence microscope. Images of *C. albicans *adhered to untreated or AC-PACs-treated epithelial cells in fluorescence mode (B and C, respectively) and in phase-contrast mode (D and E, respectively).

**Figure 5:**
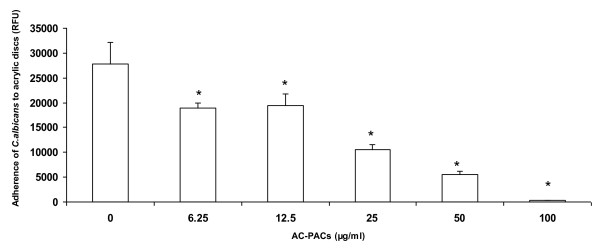
**Effect of AC-PACs on adherence of *C. albicans *to acrylic resin disks**. FITC-labeled *C. albicans *adhered to saliva-coated acrylic resin disks were quantified by measuring fluorescence in a microplate reader. Assays were done in triplicate and the means ± SD from three independent experiments were calculated. Data were performed as relative fluorescence units (RFU) obtained from the disks incubated with *C. albicans *and AC-PACs, and compared to control disks incubated with *C. albicans *alone. *, significantly lower than the value for the control (*P *< 0.05).

The effects of AC-PACs on the adherence properties of *C. albicans *to oral epithelial cells and acrylic resin discs were then tested. AC-PACs at 25 and 50 μg/ml reduced *C. albicans *adherence to oral epithelial cells by 42% ± 11% and 90% ± 14%, respectively, while a complete inhibition was observed at 100 μg/ml (Figure 4A). Fluorescence microscopy observations demonstrated a marked reduction in the number of *C. albicans *attached to epithelial cells in the presence of AC-PACs at 50 μg/ml (Figure 4C, E) as compared to untreated control (Figure 4B, D). AC-PACs were also tested for its capacity to inhibit *C. albicans *adhesion to acrylic resin discs, which represent a model for denture materials. The inhibitory effect was dose-dependent, and AC-PACs at the lowest concentration tested (6.25 μg/ml) reduced *C. albicans *adherence by 32%, while at the highest concentration tested (100 μg/ml) an almost complete inhibition of attachment of *C. albicans *to acrylic resin disks was observed (Figure 5).

To get insight onto the mechanism by which AC-PACs reduce *C. albicans *adhesion, experiments were performed to investigate whether AC-PACs can modify the cell surface hydrophobicity of *C. albicans*. A 30-min incubation of *C. albicans *with AC-PACs at a concentration of 100 μg/ml decreased the hydrophobicity index (HI) from 54% ± 4% to 7% ± 2%.

**Figure 6:**
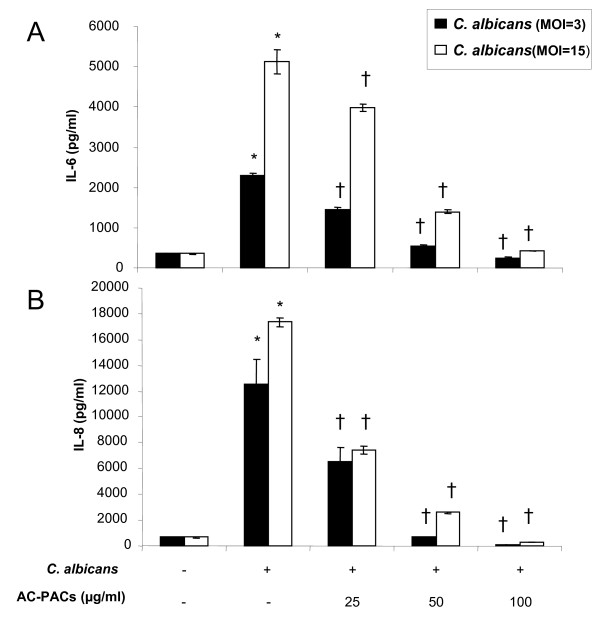
**Effect of AC-PACs on IL-6 and IL-8 secretion by *C. albicans *(MOI of 3 or 15)-stimulated oral epithelial cells**. IL-6 (Panel A) and IL-8 (Panel B) concentrations in the cell-free supernatants were determined by ELISA. Assays were run in triplicate, and the means ± SD from three independent assays were calculated. *, significantly higher than the value for the unstimulated (*C. albicans*) control (*P *< 0.05); †, significantly lower than the value for the untreated (AC-PACs) control (*P *< 0.05).

Lastly, we examined the capacity of AC-PACs to modulate the *C. albicans*-induced inflammatory response in oral epithelial cells. In this purpose, epithelial cells were pre-treated with AC-PACs prior to be stimulated with *C. albicans *cells at MOI of 3 and 15. In the absence of AC-PACs, *C. albicans *significantly and MOI-dependently induced IL-6 and IL-8 secretion by epithelial cells (Figure 6). AC-PACs decreased the secretion of both cytokines in a dose-dependent manner when epithelial cells were infected with *C. albicans *at either MOI of 3 or 15. More specifically, when epithelial cells were exposed to *C. albicans *at an MOI of 3, AC-PACs at concentrations of 25, 50, and 100 μg/ml reduced the secretion of IL-6 by 36%, 76% and 89%, respectively, as compared to control cells not treated with AC-PACs (Figure 6A). In addition, IL-8 secretion was decreased by 48%, 94% and 99%, respectively, as compared to control cells not treated with AC-PACs (Figure 6B). AC-PACs also caused a similar inhibition of secretion of both cytokines when *C. albicans *was used at an MOI of 15 (Figure 6). Neither *C. albicans *nor AC-PACs, alone or in combination, reduced the viability of epithelial cells as determined with an MTT assay (data not shown).

**Figure 7:**
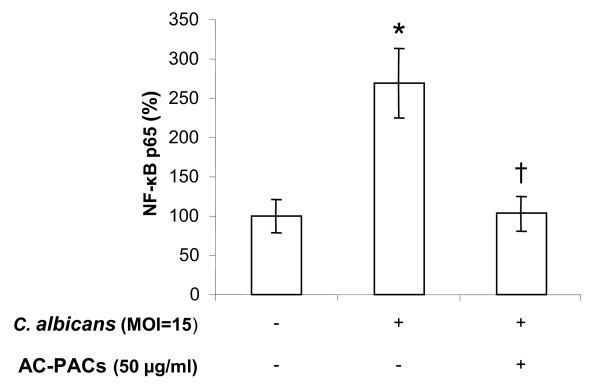
**Effect of AC-PACs on NF-κB p65 activation by *C. albicans *(MOI of 15)-stimulated oral epithelial cells**. The activation of NF-κB p65 in the cellular extract was determined by an ELISA-based assay. Assays were run in triplicate, and the means ± SD from three independent assays were calculated. *, significantly higher than the value for the unstimulated (*C. albicans*) control (*P *< 0.05); †, significantly lower than the value for the untreated (AC-PACs) control (*P *< 0.05).

The relative DNA-binding activity of nuclear transcription factor NF-κB p65 in epithelial cells infected with *C. albicans *at MOI of 15 was increased up to 290% ± 13% (Figure 7). Pretreating the cells with AC-PACs at 50 μg/ml prior to stimulating them with *C. albicans *significantly decreased the induced activity of NF-κB p65, down to the level of non-stimulated GMSM-K cells (Figure 7). Moreover, following a stimulation with *C. albicans *(MOI of 15), the levels of phosphorylated kinases, AKT (Ser473), AKT (Thr308), MEK1 (Ser217/Ser221) and ERK1/2 (Thr202/Tyr204) were significantly increased by 92%, 85%, 206% and 44%, respectively (Table 1). However, when epithelial cells were pretreated with AC-PACs at 50 μg/ml, the levels of phosphorylated AKT (Ser473) and MEK1 (Ser217/Ser221) were significantly reduced by 33% and 43% respectively, while the elevated phosphorylation of ERK1/2 (Thr202/Tyr204) returned to its basic non-stimulated state. The *C. albicans*-mediated enhanced phosphorylation level of AKT (Thr308) was not altered by AC-PACs (Table 1).

**Table 1:** Effect of AC-PACs on the levels of phosphorylated protein kinases induced by *C. albicans *in oral epithelial cells

Phosphorylated protein kinase	Amounts of phosphorylated protein kinase (pg/ml)		
	No stimulation	*C. albicans *stimulation (MOI = 15)	
		No pre-treatment	AC-PAC pre-treatment
AKT (Ser 473)	614 ± 171	1184 ± 186*	782 ± 110*
AKT (Thr 308)	60 ± 17	111 ± 29*	113 ± 24
MEK1 (Ser 217/Ser 221)	2072 ± 383	6359 ± 255*	3596 ± 773^†^
ERK1/2 (Thr 202/Tyr 204)	67 ± 2	97 ± 8*	41 ± 6^†^

The data are the means ± SD of triplicate assays. *significantly higher compared to the unstimulated control (*P *< 0.05); ^†^significantly lower compared to the untreated (AC-PACs) control (*P *< 0.05).

## Discussion

Oral candidiasis is a common fungal disease for which *C. albicans *is the major etiological agent. These infections can be controlled by several means, the most effective being a fungicidal approach. However, this approach has numerous draw backs, the most serious one being the emergence and spread of drug resistant strains [25]. A variety of virulence attributes associated to *C. albicans *are involved in the infection process. For example, the ability to adhere and to form biofilms on biomaterials [26] and oral mucosa [27] allows *C. albicans *to accumulate in large amounts. In the biofilm, *C. albicans *is protected from antimicrobial agents and the host immune system. Agents interfering with biofilm formation and adherence properties represent a novel approach to control *C. albicans *infections. By affecting *C. albicans *virulence properties, this may minimize the appearance of resistant strains. Previous studies have reported antibiofilm activities of several agents against candidal biofilms [28,29].

Proanthocyanidins isolated from the American cranberry (*V. macrocarpon*) are composed of oligomers containing at least one A-type interflavan bond, although there are often multiple A-type interflavan linkages at each degree of polymerization within the proanthocyanidin oligomeric series [20]. Numerous studies have demonstrated antiadhesion and antibiofilm properties of AC-PACs attributed to their unique A-type bond chemical structure [30-34]. While these activities of cranberry PACs have been directed against bacteria including Gram positive [33,35] and Gram negative [32,34], it is still unknown whether cranberry proanthocyanidins are able to exert such properties on eukaryotic fungi. Our study showed that the formation and architecture of *C. albicans *biofilm were strongly affected by AC-PACs. Moreover, AC-PACs were capable to detach a newly formed biofilm of *C. albicans*, a phenomenon that is likely to alter its biological functions. Importantly, AC-PACs exerted their antibiofilm activities while having no effect on fungal growth, which is in agreement with other studies reporting no alteration in bacterial growth and viability due to the presence of AC-PACs [30,34]. The use of such agents that disengage microorganisms from the biofilm without affecting their viability may prove advantageous, as selective pressure and overgrowth of resistant fungus would be avoided [36].

Our study also showed that AC-PACs inhibit *C. albicans *adherence to oral epithelial cells in a dose-dependent mode. The ability of AC-PACs to inhibit the adherence of *C. albicans *to epithelial cells suggests that this cranberry fraction may be of interest for the prevention of oral candidiasis. Indeed, adhesion to epithelial cells is a key event in pathogenic lifestyles of *C. albicans *[37,38] and consequently its prevention could minimize the fungal virulence. *C. albicans *has been recognized as the primary agent of denture-related stomatitis, an inflammatory process affecting the oral mucosa of 30-60% of patients wearing removable dental prostheses [39,40]. AC-PACs also significantly reduced *C. albicans *adherence to acrylic resin discs that mimic denture material.

Hydrophobic interactions are generally considered to play an important role in the adherence of *C. albicans *to eukaryotic cells and also to certain inert surfaces, such as prosthetic devices [41]. Therefore, agents able to modify surface characteristics of *C. albicans *may alter their adherence capacity, and subsequently prevent biofilm formation and subsequently invasion of host cells. In the present study, AC-PACs markedly modified *C. albicans *cell surface from being highly hydrophobic to hydrophilic. This is in agreement with Ishida et al. [24], who reported that tannins isolated from *Stryphnodendron adstringens *decrease adherence of *C. albicans *to mammalian cells and to glass surfaces by lowering the fungi cell surface hydrophobicity [24]. Thus, we propose that the mechanism of antiadhesion and antibiofilm action of AC-PACs may be at least in part attributed to a modification of *C. albicans *cell surface. Previous reports documented a strong correlation between inhibition of bacterial biofilm formation by various cranberry constituents and reduction of hydrophobicity of bacterial membrane [42,43]. Cranberry PACs have been shown to alter specifically and non-specifically bacterial surface macromolecules (fimbriae, LPS) [44]. Interestingly, exposure of bacteria to cranberry juice for even a short time period produced important changes in surface composition [45]. Therefore, in order to evaluate the exact mode of antiadhesion activity, further experiments are required to determine the effect of AC-PACs on *C. albicans *cell wall-associated adhesins such as ALS, Hwp1, Eap1, Iff, which mediate fungal adherence [5,27,46].

The tissue homeostasis is regulated by several host factors produced by immune and mucosal cells in a healthy condition. Production of proinflammatory mediators by host cells in response to *C. albicans *plays a critical role in the activation of immune cells and final clearance of the organism [47]. Infections of oral epithelial cell models with *C. albicans *identified a wide range of pro-inflammatory mediators for which the secretion was markedly increased [48]. In the present study, oral epithelial cells infected with *C. albicans *(MOI = 15) secreted a dramatically increased amount of the pro-inflammatory cytokines, IL-6 (15-fold) and IL-8 (25-fold). IL-6 and IL-8 are considered as the major pro-inflammatory cytokine and chemokine for the recruitment and activation of neutrophils and macrophages at the site of infection [49,50]. However, due to the protecting reaction of the host against fungal pathogens, an accumulation of inflammatory mediators occur and may lead to a chronic and persistent inflammation, and ultimately to tissue destruction mediated by MMPs. In regard to the resolution of the inflammatory process, prevention of excessive activation of innate immunoeffectors or timely inactivation of their function is critical for recovery from fungal infection. We showed a dose-dependent inhibitory effect of AC-PACs on *C. albicans*-induced secretion of IL-6 and IL-8 by epithelial cells. These findings are in agreement with previous studies reporting a strong anti-inflammatory activity of proanthocyanidin-rich cranberry fractions towards different human cell lines infected with pathogens [34,51-53].

The mechanisms involved in the proinflammatory mediator overexpression mediated by *C. albicans *in human keratinocytes includes toll-like receptor-2 (TLR-2)-induced activation of intracellular signal transduction pathways including NF-κB and various kinases [54]. In the present study, we observed a significant activation of NF-κB p65 as well as an increased phosphorylation of four kinases, AKT (Ser473), AKT (Thr308), MEK 1(Ser217/Ser221) and ERK 1/2 (Thr202/Thr204) in oral epithelial cells infected with *C. albicans*. Interestingly, stimulation of cytokine production in human monocytes by proteinases of *C. albicans *was suggested to be regulated via AKT/NF-κB pathway, where AKT initiates translocation of NF-κB into the nucleus [55]. In addition, it has been shown that inflammatory response of synovial fibroblasts induced by *C. albicans *involves activation of ERK 1/2 and is associated with NF-κB activation [56]. The enhanced activation of extracellular signal-regulated kinases and suppression of arachidonic acid release in *C. albicans*-infected macrophages were reduced by MEK1 inhibitor, suggesting that this kinase plays important role in candidiasis-associated inflammatory processes [57]. We provided evidence that AC-PACs inhibited, at least in part, the *C. albicans*-induced secretion of IL-8 and IL-6 in oral epithelial cells by their ability to inhibit the activation of NF-κB p65. This is in agreement with previous studies showing that cranberry proanthocyanidins exert their anti-inflammatory properties by modulating the NF-κB pathway [19,34,58]. Moreover, the present study showed that AC-PACs inhibit the phosphorylation of major intracellular signaling proteins induced by *C. albicans*. Thus, it is suggested that AC-PACs by interfering in signal transduction cascade may contribute to reduce the impact of host inflammatory processes mediated by elevated production of IL-6 and IL-8 occurring in oral candidiasis.

## Conclusion

The present study demonstrated that AC-PACs, by affecting the virulence properties of *C. albicans *and, parallely, attenuating the inflammation induced by this pathogen, may have a beneficial effect as a novel therapeutic in prevention and treatment of oral candidiasis. Clinical trials are required to demonstrate whether the beneficial properties demonstrated under our assay conditions may be observed *in vivo*.

## Competing interests

The authors declare that they have no competing interests.

## Authors' contributions

All authors contributed equally in data acquisition and in writing of the manuscript. All the authors read and approved the final version of the manuscript.

## Pre-publication history

The pre-publication history for this paper can be accessed here:

http://www.biomedcentral.com/1472-6882/12/6/prepub
